# Women’s Midlife Health reviewer acknowledgement 2015

**DOI:** 10.1186/s40695-016-0015-1

**Published:** 2016-02-18

**Authors:** Siobán Harlow

**Affiliations:** University of Michigan, 500 S State St, Ann Arbor, MI 48109 USA

## Abstract

The editor of *Women’s Midlife Health* would like to thank all of the reviewers who have contributed to the journal in Volume 1 (2015).

**Annemarie Koster**

Netherlands

**Carrie Karvonen-Gutierrez**

United States of America

**Cassandra Szoeke**

Australia

**Elsa Strotmeyer**

United States of America

**Gail Greendale**

United States of America

**Gary Venolini**

United States of America

**Geena Athappilly**

United States of America

**Glinda Cooper**

United States of America

**Gloria Bachmann**

United States of America

**Janis Miller**

United States of America

**Jodi Flaws**

United States of America

**John Randolph**

United States of America

**Josefina Romaguera**

Puerto Rico

**Joyce Bromberger**

United States of America

**L. Elaine Waetjen**

United States of America

**Lorraine Dennerstein**

Australia

**Martha Goetsch**

United States of America

**Mary Sammel**

United States of America

**Michelle Warren**

United States of America

**Morgana Mongraw-Chaffin**

United States of America

**Nancy Phillips**

United States of America

**Nancy Woods**

United States of America

**Siobán Harlow**

United States of America

